# Genome-Wide Discovery of Small RNAs in *Mycobacterium tuberculosis*

**DOI:** 10.1371/journal.pone.0051950

**Published:** 2012-12-19

**Authors:** Paolo Miotto, Francesca Forti, Alessandro Ambrosi, Danilo Pellin, Diogo F. Veiga, Gabor Balazsi, Maria L. Gennaro, Clelia Di Serio, Daniela Ghisotti, Daniela M. Cirillo

**Affiliations:** 1 Emerging Bacterial Pathogens Unit, S. Raffaele Scientific Institute, Milan, Italy; 2 Dipartimento di BioScienze, University of Milan, Milan, Italy; 3 University Statistical Center for Biomedical Sciences – Università Vita-Salute S. Raffaele, Milan, Italy; 4 Department of Systems Biology, The University of Texas MD Anderson Cancer Center, Houston, Texas, United States of America; 5 Public Health Research Institute, University of Medicine and Dentistry of New Jersey, Newark, New Jersey, United States of America; National Institute for Infectious Diseases (L. Spallanzani), Italy

## Abstract

Only few small RNAs (sRNAs) have been characterized in *Mycobacterium tuberculosis* and their role in regulatory networks is still poorly understood. Here we report a genome-wide characterization of sRNAs in *M. tuberculosis* integrating experimental and computational analyses. Global RNA-seq analysis of exponentially growing cultures of *M. tuberculosis* H37Rv had previously identified 1373 sRNA species. In the present report we show that 258 (19%) of these were also identified by microarray expression. This set included 22 intergenic sRNAs, 84 sRNAs mapping within 5′/3′ UTRs, and 152 antisense sRNAs. Analysis of promoter and terminator consensus sequences identified sigma A promoter consensus sequences for 121 sRNAs (47%), terminator consensus motifs for 22 sRNAs (8.5%), and both motifs for 35 sRNAs (14%). Additionally, 20/23 candidates were visualized by Northern blot analysis and 5′ end mapping by primer extension confirmed the RNA-seq data. We also used a computational approach utilizing functional enrichment to identify the pathways targeted by sRNA regulation. We found that antisense sRNAs preferentially regulated transcription of membrane-bound proteins. Genes putatively regulated by novel *cis*-encoded sRNAs were enriched for two-component systems and for functional pathways involved in hydrogen transport on the membrane.

## Introduction

Regulatory RNA species can modulate transcription, translation, mRNA stability, DNA maintenance, and gene silencing. They function through a variety of mechanisms, including changes in RNA conformation, protein binding, base pairing with other RNAs, and interaction with DNA [1]–[3]. It has been established that small RNAs (sRNAs) are crucial elements regulating gene expression in Gram-positive and Gram-negative bacteria [4]–[7]. The prototype of a bacterial sRNA is a non-coding RNA of 50–300 nucleotides (nt) in length that acts by imperfect base pairing with *trans* encoded RNA target(s). sRNAs may also be *cis*-encoded and transcribe antisense (AS) to the target RNA. Recent research has shed some light on the relevance of sRNAs in bacterial pathogenesis, including modulation of expression of virulence factors in response to environmental and host signals [2], [8]–[14].

Intracellular survival of *Mycobacterium tuberculosis* (MTB) results from the ability of this pathogen to sense the host environment and to switch to a non-replicating, persistent form that is resistant to host insult and to treatment with most antibiotics. Multiple genes associated with persistence have been identified in MTB. However, the complex post-transcriptional regulation underlying the phenotypic switch to persistence remains poorly understood. In particular, our knowledge of the regulatory networks involving sRNAs is still in its infancy. Understanding how sRNAs control bacterial virulence may open a new prospective in the fight against tuberculosis (TB) and contribute to the goal of TB eradication [15]–[18].

About 40 species of sRNAs have been reported so far in mycobacteria (including MTB) by cloning approaches and computational predictions [19], [20]. Additional sRNAs have been identified by RNA sequencing (RNA-seq) [21], [22]. RNA-seq results for sRNAs however heavily depend on threshold definition for reads coverage and on the criteria used for transcript annotation, thus requiring further validation [3]. Here we report the first genome-wide expression analysis of sRNAs in MTB by microarray followed by size determination, mapping and computational target prediction.

## Results

### Genome-wide Expression Profiling of sRNAs in MTB

To identify sRNAs in MTB, we performed microarray expression analysis on small-size RNA enriched fraction extracted from exponentially growing cultures of *M. tuberculosis* H37Rv. We used a set of 1373 sRNA species previously identified by RNA-seq (Type A candidates, [22]), which was updated to reflect changes in annotation in the genome of MTB (type A, Table S1).

**Figure 1:**
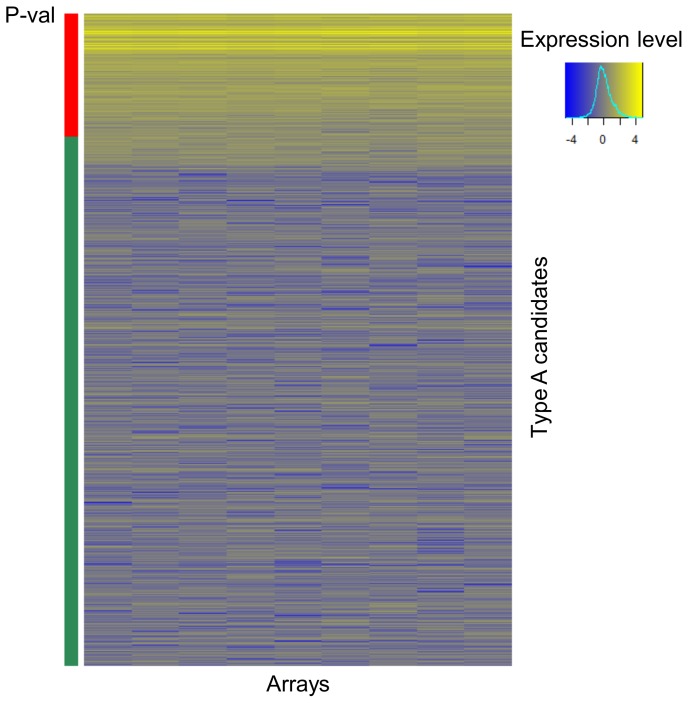
Heat-map representing the expression profiles of microarrays. As positive controls we used reference *sigA*, *rrs* and the short ribosomal 5S RNA. Normalized control genes showed a mean expression level of 7.16±1.57. Expression level: yellow to blue (highly expressed to lowly expressed). Statistics: red = p-value <0.05; green = p-value >0.05.

For microarray analysis, we considered as expressed only the candidates showing an expression mean significantly higher (p-value <0.05) than the positive control genes (this group included genes expressed during exponential growth such as *sigA* and *rrs* and the short ribosomal 5S RNA; for a complete list of control genes, see Materials and Methods). The microarray analysis validated 258 (18.8%) species (Figure 1), about a third of which (n = 97) showed a length below 50 nucleotides. When we calculated the minimum free energy (MFE) associated p-value, which represents a measure of secondary structure stability, we found that the proportion of sRNAs showing an MFE-associated p-value <0.05 was nearly double for “array-validated” candidates (37/258, 14.3%) than for the remaining “non validated” candidates (85/1115, 7.6%).

### Classification of MTB Putative sRNAs

A functional classification of the selected sRNAs identified was performed based on genome location (see Materials and Methods and Figure S1). We found 22 intergenic sRNAs, 84 candidate sRNAs mapping within 5′ or 3′ UTRs, and 152 antisense sRNAs (Table 1).

**Table 1:** Classification of validated sRNAs according to their genomic position.

Category	Number (%)
Intergenic	22 (8.5)
5′/3′ UTR	84 (32.6)*
AS	99 (38.4)
AS to 5′/3′ UTR	53 (20.5)
Total	258 (100.0)

UTR: untranslated region; AS: antisense.

* 5′ UTR: 51; 3′ UTR: 8; both 5′ and 3′ UTR: 25.

The two more represented classes identified by microarrays were AS RNAs (60%) and 5′/3′ UTRs (32.6%); of the latter class, most were 5′UTRs (5′UTRs: 90%). RNA species identified within the 5′ UTRs could represent *cis*-acting regulatory elements such as riboswitches. Between the 5′UTRs we found genes involved in cell wall processes, intermediary metabolism, information pathways, PE/PPE, virulence, detoxification and adaptation, and lipid metabolism (Table S2).

### Statistical Analysis of the Frequencies of Specific Nucleotides at the −10 Region

To show that the different RNAs validated by microarray expression analysis were transcribed, we compared the frequency of the nucleotide’s distributions at specific positions within the −10 region between sRNA candidates, annotated coding sequences (CDSs), and random sequences. We could confirm the effective transcription of sRNAs identified by microarrays by showing a clear non-random distribution of nucleotides within the −10 region. In particular, we showed that our candidates show a −10 region more similar to the one of CDSs rather than to random genomic sequences not annotated for transcripts. To achieve this goal we used a statistical approach previously described by Dornenburg [23]. We compared the frequency of nucleotides at the −10 consensus hexamer between sRNA candidates, annotated CDSs, and random sequences. The hexamer TANNNT recognized by the sigma factor A was used as reference consensus. For the set of annotated CDSs, −10 hexamers match the consensus, on average, 1.7 times out of 3 known consensus nucleotides −10 match score). In contrast, randomly selected sequences match the consensus only 1.5 times out of 3 (control match score) (Figure S2). Due to the high number of terms compared for each category (see Materials and Methods), this difference is highly significant (P<0.0001). The −10 match score for the sRNAs is not significantly different from that of the set of CDSs. The comparison of Type A candidates only to the control group of random sequences and to the consensus sequences of annotated CDSs showed similar results. Considering microarray-validated candidates only (score 2.0), the difference with the dataset from CDSs increased whereas that with the random sequences slightly decreased.

### Characterization of sRNA by Northern and Identification of the 5′-end by Primer Extension

Microarray data were further validated by Northern blot analysis. We selected 23 candidate sRNAs (Table 2) from all the functional categories identified (see Table 1). Genomic mapping of these sRNAs is shown in Figures S3 and S4.

The candidates chosen were:

four intergenic (#149, 161, 224 and 1096);
eight 5′/3′ UTRs of genes (#1149, 1414, 1502, 1542, 1565, 106, 189, and 193);
ten AS (#54, 135, 1029, 540, 1080, 1556, 488, 1137, 294, and 498).

We also included candidate 1359, although it was mapped within the *Rv2165c* gene, since it is highly expressed by microarray analysis. As a negative control we used a 5′UTR sRNA not validated by microarray (#76). Since Northern blot analysis allows relative comparison of transcript abundance, RNA was extracted from exponential and stationary phase cultures in order to evaluate differential expression associated with growth state. Total RNA was extracted from the strain H37Rv. For a smaller pool (candidates #224, 540, 1137, and 1565) we also tested the attenuated strain H37Ra.

**Table 2:** Validation of 23 selected candidates by Northern blot.

ID	Start	End	Strand	RNA-seq	Northern^a^	−10	Stem-loop	Mapping
54	498401	498455	+	54	75		Yes	AS to Rv0412c^b^
76^d^	629877	629975	+	98	Neg			5′ UTR, Rv0538
106	806185	806218	+	33	Neg			3′ UTR, Rv0710
135	1040645	1040938	+	293	45, 250	sigA	Yes	AS to Rv0932c
149	1200555	1200605	+	50	75			Intergenic
161^e^	1287126	1287201	+	75	180, 160	sigA	Yes	Intergenic
189	1453007	1453060	+	53	50	sigA	Yes	3′ UTR, Rv1296
193^e^	1476825	1476884	+	59	70, 35			3′ UTR, Rvnr02 or 5′ UTR, Rvnr03
224^b^	1735693	1735747	+	54	300, 90			Intergenic
294	2268166	2268257	+	91	70	sigA		AS to 3′, Rv2023A or 5′, Rv2023c
488^e^	3557057	3557335	+	278	>300, 280, 130	sigA^h^	Yes	AS to 3′ UTR, Rv3191c
498^e^	3597808	3598107	+	299	150	sigA	Yes^g^	AS to 3′, Rv3221A or 5′, Rv3221c
540 f	4025441	4025570	+	129	70			AS to 5′ UTR, Rv3583c
1029	732392	732787	–	395	Neg	sigA		AS to Rv0636
1080^e^	937956	938239	–	283	230	sigA		AS to 5′, Rv0842
1096	1041129	1041165	–	36	300, 120, 80, 65, 60, 55	sigA	Yes	Intergenic
1137^f^	1275611	1275673	–	62	40	sigA	Yes	AS to 3′, Rv1147
1149	1306038	1306073	–	35	80,70, 30	sigA	Yes	5′ UTR, Rv1174c
1359e,c	2429342	2429373	–	31	60, 30	sigB		5′ UTRs, Rv2165c
1414	2641081	2641126	–	45	Neg			5′ UTR, Rv2357c
1502^e^	3363023	3363153	–	130	100, 80, 50	sigA	Yes	5′ UTR, Rv3003c
1542^e^	3621265	3621466	–	201	280, 60	sigA		5′ UTR, Rv3241c
1556^e^	3800005	3800091	–	86	30	sigA		AS to 5′, Rv3386
1565^f^	3837288	3837458	–	170	500, 160, 90, 60	sigA	Yes	5′ UTR, Rv3418c

a Length of the principal bands visualized by Northern blot analysis.

b The presence of a −10 sigA consensus sequence about thirty-eight nucleotides upstream of the predicted 5′end suggests that the sRNAs length is in accordance with the band observed by Northern blot.

c Type C candidate.

d Negative control.

e Northern blot tested in *M. tuberculosis* H37Rv, exponential and stationary growth phases.

f Northern blot tested in *M. tuberculosis* H37Rv and *M. tuberculosis* H37Ra (exponential growth phase).

g considering 3′-end mapping according to Northern blot results (175 nucleotides downstream the predicted 3′-end).

h considering the 5′-end mapping according to the 5′-end primer extension.

**Figure 2:**
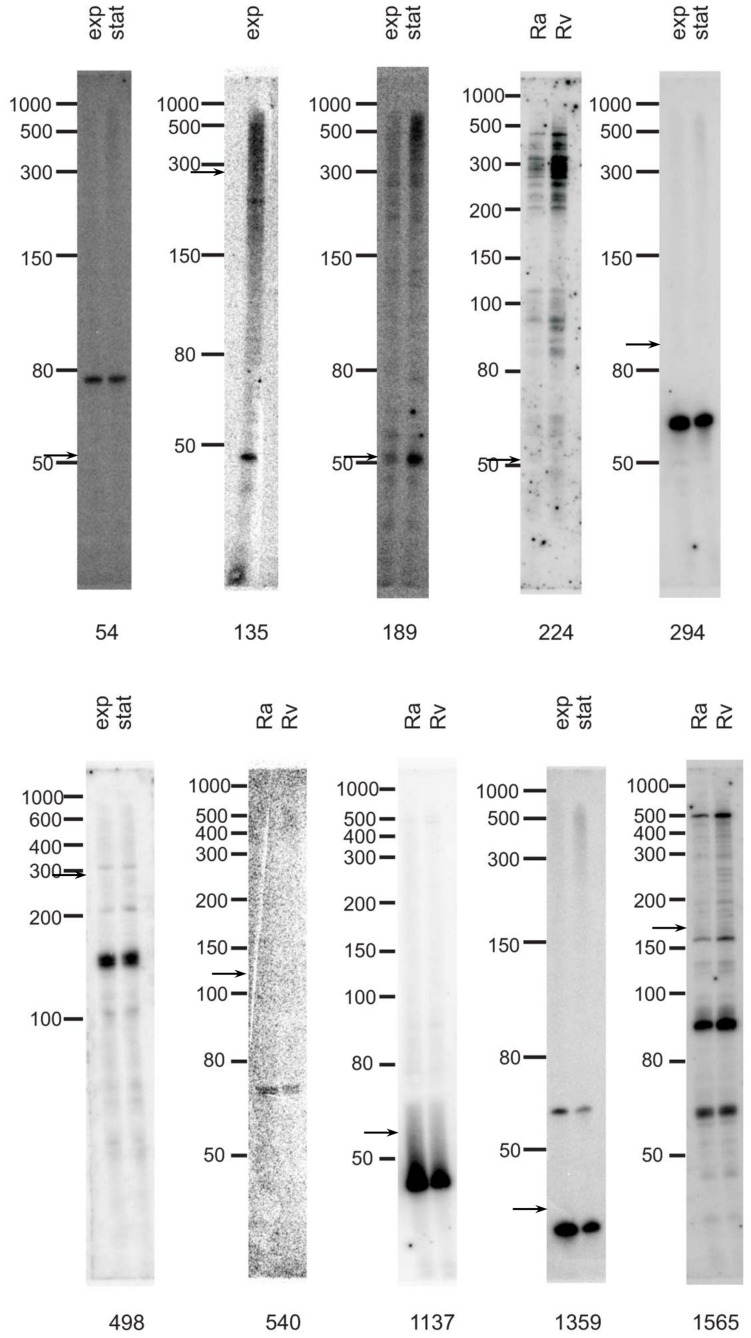
Northern blot results for selected candidates. The RNAs were extracted as indicated in M&M: exp: *M. tuberculosis* H37Rv in exponential growth phase; stat: *M. tuberculosis* H37Rv in stationary growth phase; Rv: *M. tuberculosis* H37Rv; Ra: *M. tuberculosis* H37Ra. Arrows indicate the predicted length.

The results of the Northern blot showed that in most cases (20 out of 23), one or more specific bands were detected (Table 2, and Figure 2). The Northern blot of candidate #189 showed a single band of predicted length; in other cases (#1565) a band of the predicted size was detected among several others. For most candidates, however, the length observed by Northern analysis differed from the results of the RNA-seq-based computational mapping (for example, Figure 2, #498 and 1137). sRNAs #106, 1029 and 1414 and the negative control #76 were negative by Northern blot analysis (data not shown). No difference was observed between exponential and stationary phase expression for all sRNAs cultures (Figure 2). Only candidate #224was expressed at higher levels in *M. tuberculosis* H37Rv than in H37Ra.

Since computationally predicted length and that observed by Northern blot analysis differed in almost all species, we set out to identify the 5′ ends of eight selected sRNAs (#488, 498, 1080, 1137, 1149, 1359, 1502 and 1565) by primer extension. In all cases, we could identify one or more bands, one of which corresponded to the predicted 5′ end with +1 or −1 nt difference (data not shown). A −10 consensus sequence for sigma factor A (TANNNT) was identified for 7 out of 8 sRNAs mapped, suggesting that transcription of the sRNAs is under sigA control. Upstream of the candidate 1359 a good consensus sequence for −10 and −35 sigma B is present (NGTGG – N_14–18_–NNGNNG).

### Identification of the −10 sigA Consensus Sequences and Putative Terminators in sRNA Candidates

Since the 5′ ends identified by primer extension well matched those predicted by RNA-seq, the discrepancies between the length observed by Northern blot and computational analysis might mainly be due to inaccurate 3′-end mapping. First, we performed the analysis of all the sRNAs upstream sequences to detect the presence of −10 consensus for sigA. We decided to search for sigA consensus motifs because this is the primary sigma factor that regulates gene expression during the exponential growth phase. We searched for −10 sigma A consensus sequences (TANNNT) within −50 and +15 nucleotides from the 5′-end mapped according to RNA-seq results. At the same time, we searched for putative terminators downstream of the predicted sRNAs. The 3′-end region (−20/+270 nucleotides) was sought for intrinsic terminators using the GeSTer algorithm [24], and for rho-independent terminators, according to Gardner *et al.* [25]. In 60.5% of cases we detected a sigA promoter consensus sequence upstream of the presumed 5′-end. Terminator consensus motifs were present in 22.1% of the regions downstream of the sRNA. Only a minor percentage (13.6%) of sRNAs presented both promoter and terminator sequences, whereas one third (31.0%) of predicted candidates did not show consensus motifs either for sigma A promoters or terminators (Table 3).

**Table 3:** Promoter and terminator consensus sequences found among 258 *M. tuberculosis* sRNAs by bioinformatics approach.

Consensus	n (strand+; strand–)	%
promoter POS; terminator POS	35 (6; 29)	13.6
promoter POS; terminator NEG	121 (60; 61)	46.9
promoter NEG; terminator POS	22 (8; 14)	8.5
promoter NEG; terminator NEG	80 (42; 38)	31.0
Total	258 (116; 142)	100.0

Sequence inspection of the −50/+15 region at the predicted 5′end for sigA consensus sequences and of the region −20/+200 at the 3′ end for termination signals. Presence (POS); Absence (NEG).

Classification of all 1373 candidates, promoter and terminator consensus sequence analyses, and genomic annotation are summarized in Table S1.

### *In silico* Identification of Pathways Regulated by Antisense sRNAs

An important question in sRNA biology is to identify the genes that these molecules regulate. We performed an *in silico* analysis to identify pathways regulated by the set of novel sRNAs discovered in MTB. We focused on AS RNAs because the direct targets for this class of sRNAs are known. When we performed microarray analysis we found 152 AS sRNAs that overlap the same number of unique target genes in the opposite strand (Table 1). Using functional enrichment analysis, we identified molecular functions and pathways enriched in AS-regulated genes in MTB. In particular, hydrogen transport on the membrane (GO:0015078, GO:0015077, GO:0006818, GO:0015992, GO:0034220) and two-component systems (mtu02020) emerged as function potentially subjected to antisense regulation. When we analyzed cellular location, we found that membrane-bound proteins were preferentially targeted (GO:0044425, GO:0005886, GO:0031224). These results are summarized in Table 4. The corresponding targeted genes can be found Table S3.

**Table 4:** Functional enrichment of *cis-*regulated genes.

Term	Description	N Genes	p-value
GO:0006796	phosphate metabolic process	7	0.0066
GO:0015078	hydrogen ion transmembrane transporter activity	4	0.0097
GO:0043284	biopolymer biosynthetic process	13	0.0117
GO:0019538	protein metabolic process	15	0.014
GO:0044271	nitrogen compound biosynthetic process	2	0.0153
GO:0006793	phosphorus metabolic process	7	0.0156
GO:0044425	membrane part	26	0.0178
GO:0015077	monovalent inorganic cation transmembrane transporter activity	4	0.0187
GO:0005886	plasma membrane	16	0.0194
GO:0046933	hydrogen ion transporting ATP synthase activity, rotational mechanism	3	0.0208
GO:0031224	intrinsic to membrane	25	0.022
GO:0045259	proton-transporting ATP synthase complex	3	0.0266
GO:0016020	Membrane	27	0.0308
GO:0006818	hydrogen transport	3	0.0335
GO:0015992	proton transport	3	0.0341
GO:0016310	Phosphorylation	6	0.037
GO:0044267	cellular protein metabolic process	11	0.0389
GO:0051171	regulation of nitrogen compound metabolic process	4	0.0405
GO:0015986	ATP synthesis coupled proton transport	3	0.0442
GO:0016469	proton-transporting two-sector ATPase complex	3	0.0442
GO:0015985	energy coupled proton transport, down electrochemical gradient	3	0.0449
GO:0010556	regulation of macromolecule biosynthetic process	4	0.0457
GO:0034220	ion trans-membrane transport	3	0.0491
mtu02020	two-component system	6	0.0363
mtu00230	purine metabolism	6	0.0732

Functional annotation of *M. tuberculosis* genes was obtained from different databases; Fisher’s exact test, P<0.05 (see Methods).

GO: gene ontology; mtu: KEGG pathway.

## Discussion

Here we report on the integration of experimental and computational methods to identify 258 novel antisense and intergenic sRNAs in MTB. The present work derives from a previous, genome-wide identification of candidate sRNAs by RNA-seq [22]. Large RNA-seq dataset require accurate validation, especially for genome annotation of novel transcripts. We considered reliable the sRNAs identified with high reads coverage and validated these sRNAs by microarray and, for representative species of the microarray-validated pool, by Northern blot and primer extension analyses. This approach provided more details on the annotation of the candidates; indeed, the particular design strategy we adopted for microarray probes allowed acquiring important data on the size of the sRNAs. The inclusion of probes targeting well-known control genes permitted the collection of expression data for these newly discovered sRNAs.

Our results lead to several conclusions. As supported by −10 region and 5′ end mapping analyses, our computational approach applied to Illumina sequencing results provided reliable detection of sRNA transcripts. All but one of the sRNAs mapped experimentally are preceded by a sigA promoter consensus sequence. In addition, an initial analysis of sRNA target pathways indicated that AS sRNAs preferentially target genes involved in two-component systems and membrane activity. Our results suggest that expression of the sRNAs described may be not as part of the stress response, but are actively involved in the regulation of basic metabolism in MTB.

Together, our study provides a comprehensive top-down demonstration of methods whereby large sets of RNA-seq data on sRNAs can lead to detailed sRNA mapping and identification of target pathways. Our analytical path differs from previous studies on sRNAs of *M. tuberculosis*, which reported only few sRNAs and did not provide a prediction of putatively regulated pathways. Indeed, despite Arnvig and colleagues performed RNA-seq to identify novel sRNAs in MTB [21], they provided an overview on genome-wide data and focused their attention characterizing only few of the sRNAs detected. In the present report we further characterized several candidates previously identified by us (e.g. #84, #1169, #1137, #488) [22] and by Arnvig and colleagues (MTS0479, MTS0997, MTS0903, MTS2458, respectively) [21]. Our work provides more comprehensive data on recently discovered sRNAs in MTB and statistics analysis support our experimental data: in fact, the genomic regions where we mapped sRNA candidates showed consensus sequences for active transcription indicating the probability to be transcribed.

Our starting dataset included a high number of candidates (>1000) as reported by other studies based on RNA-seq approach [26]. By microarray expression profiling, we validated nearly 20% of the previously identified putative sRNAs. These sRNA species showed to be enriched in higher secondary structure stability, as supported by MFE analysis. We found ∼100 sRNAs shorter than 50 nucleotides. sRNAs are usually between 50 and 200 nt in length; however, smaller RNAs of the size of microRNAs have been described in bacteria [23], [27]. The functional significance of these bacterial microRNAs remains to be elucidated.

Most of the sRNAs identified are antisense. This is in agreement with recent findings that extensive antisense transcription occurs in bacteria, as it does in *Eukarya* and in *Archaea* [14], [23], [28]–[31]. The target gene for AS sRNAs is likely to be the antisense CDS, whose expression is negatively controlled either by interfering with transcription/translation or by favoring mRNA degradation. The analysis of pathways regulated by AS sRNAs identified enrichment for two-component systems and cell-wall components. This preliminary finding suggests that *cis-*acting RNAs may inhibit stress-responsive modules, such as the two-component systems [32]–[34], and they may also affect other critical functions such as the metabolism of the cell wall, which is a virulence determinant of the tubercle bacillus [35]–[37].

We have hypothesized a possible function for some of the 5′/3′ UTR sRNAs validated by Northern blot. Some of our sRNAs have a role in transcription attenuation: candidate 1502, upstream of gene *ilvB* (Figure S4), coincides with the previously described *mcr9-mpr14* [20]. However, while DiChiara *et al.* detected this sRNA only in *Mycobacterium bovis* BCG, we detected *mcr9-mpr14* in MTB H37Rv. Since the *ilvB* gene is part of the isoleucine-valine operon, the presence of the short peptide encoded by the candidate 1502 (with its typical structure “3 valine, 1 isoleucine”) suggests that also in mycobacteria, as in most bacteria investigated so far [38], this sRNA is part of an attenuator system for controlling valine-isoleucine synthesis. In order to confirm the existence of attenuation in mycobacteria, we analyzed the sRNA candidate that was mapped upstream of *leuA*. This candidate (#1938; see Table S1) was classified among class C sRNAs [22] and was not included in our initial selection. By sequence analysis we found a 13 amino acids long coding region, containing a stretch of 3 consecutive leucine codons, and followed by a stem-loop structure suggesting a possible role in the control of *leuA* expression. The identification by Northern analysis of multiple bands at 85, 65 and 45 nt, respectively (Figure S5) is in good agreement with an attenuator role of this candidate. Interestingly, this sRNAs is the only species tested in this work that showed differential relative signal intensity between virulent and avirulent *M. tuberculosis* strains (Figure S5), suggesting a different control of this biosynthetic pathway in these two mycobacteria.

Our work suggests the identification of alternative RNA-based regulatory tools such as riboswitches within the 5′-end of mRNAs. Riboswitches are part of the mRNA molecule they regulate, usually the 5′UTR, and hence act in *cis*. They can adopt different conformations in response to environmental signals, including stalled ribosomes, uncharged tRNAs, elevated temperatures, or small molecule ligands [39]. Thus, riboswitches include the above-mentioned attenuators. In accordance with the findings of Arnvig *et al.* [21], our data suggest that in MTB this kind of regulation is rather widespread, as it was found in other Gram-positive bacteria like *B. subtilis* [40], [41].

Arnvig and colleagues used the reads-coverage ratio between the 5′ UTR and the downstream CDS to better identify putative riboswitches [21]. We performed RNA-seq only on the small-size RNA fraction, therefore the comparison of reads-coverage between candidates and CDSs could not be performed. However, by combining our experimental observations and computational analysis we could detect some putative riboswitches.

Indeed, our data suggest that candidate 1149 might be co-transcribed with *TB8.4* and could act as a riboswitch, regulating *TB8.4* expression, encoding a low molecular weight T-cell antigen [42] The candidate 1096 could also be a riboswitch regulating *pstS2*, one of the three components of the inorganic phosphate detection component of the membrane-associated phosphate-specific transporter (Pst) [43], [44]. *pstS*2 knockout strains showed attenuated virulence [44]. Moreover, *pstS2* and *pknD* genes are encoded within the same operon, and *pknD* knockout strains do not survive in inorganic phosphate poor-growing conditions [43]. Thus, candidate 1096 may have a crucial sensing role in controlling the *pstS2-pknD* operon. Interestingly, the candidate 135 mapped antisense to the 5′ region of the *pstS2* gene (Figure S3). Thus, the operon encoding for this gene seems to be under tight control.

Other putative riboswitches we have identified are candidate 161, controlling expression of a nitrate reductase (*narGHJI* operon) [45], [46] (Figure S3) and candidate 1565, mapping within the 5′UTR of *groES*, encoding for a co-chaperonin protein [47]. Arnvig and colleagues [21] identified the expression profile for the 5′ UTR associated with the *groES* gene. We suggest that this sRNA may be a thermosensor, as frequently found in other bacteria upstream of genes with a function similar to *groES*.

Several predicted intergenic sRNAs have been confirmed by Northern analysis (149, 161, 224 and 1096 in Table 2). A more careful analysis of other sRNAs indicated that some of them were likely to be also intergenic. This is the case for candidate 1359. Looking at the annotated sequence we observed that this sRNA maps within *Rv2165c* (*mraW*, S-adenosyl-methyltransferase). However, by comparing the polypeptide encoded by MTB with the protein encoded by *M. bovis* BCG we found that homology starts 53 amino acids downstream of the indicated start codon, where a GTG codon is present (nucleotide 2429447) preceded by a Shine-Dalgarno sequence (GGGGAGG) (Figure S6). Thus, candidate 1359 is transcribed upstream of *Rv2165c*, in the intergenic region.

Candidate 488, previously reported by Arnvig *et al.* (MTS2458; [21]), and originally classified in this work as antisense to 3′UTR of *Rv3191c*, may be intergenic since it was not overlapped to the 3′end of the *Rv3191c*, as supported by the length of the transcript visualized on Northern blot.

Candidate 498 is transcribed in opposite direction to both neighboring genes *TB7.3* and *Rv3321A*. Its predicted length (300 nt) classified this sRNA as a 3′UTR to *Rv3321A*. However, the length observed by Northern blot, the presence of both a sigma A consensus sequence and a possible stem-loop structure support the hypothesis that the candidate 498 is a *trans-*acting sRNA, whose targets have to be identified, rather than a sRNA overlapping to the *Rv3321A*-3′ end.

Candidate 190 mapped upstream of 16S rRNA gene being entirely contained in *mcr3* sRNA gene [20]. Interestingly, upstream of the 5′ end of *mcr3* no promoter consensus sequences were present. On the contrary, upstream of the 5′end of 190, which is internal to *mcr3*, we found −10 and −35 sigA consensus sequences, that can be identified with the most active rRNA operon promoter, P3 [48]–[50]. Thus sRNAs such as 190, 191, and 192 mapping in the ribosomal DNA could be *cis*-acting regulatory elements controlling unique MTB rRNA operon.

The candidate 193 (predicted length: 60 nucleotides) mapped between the ribosomal RNAs 23S and 5S (*rrl* and *rrf* genes, respectively). According to the positive signal obtained by Northern blot, this putative sRNA might be a residual RNA derived from ribosomal RNA processing. Additional bands at higher and lower molecular weight observed by Northern blot support this hypothesis. Very recently, ribosomal noncoding RNAs have been described in eukaryotic cells [51]. Despite similar mechanisms have not been described in bacteria, further experiments are needed to confirm the small RNA nature of this candidate originating by the ribosomal genomic region.

Overall, our data greatly increase the complexity of sRNA complement in MTB and suggest that RNA-mediated regulation in this organism may be as common and multifaceted as in other bacteria [3], [52]. Furthermore, we detected several new sRNAs and riboswitch-like regulators, which provide new insights into the dynamic regulation of gene expression in MTB. Indeed, riboswitches represent a cost-effective means for genetic regulation compared with protein synthesis and they also provide an immediate feedback response.

## Materials and Methods

### Ethics Statement: N/A

#### RNA extraction

For analysis of sRNAs, *M. tuberculosis* H37Rv was grown in Middlebrook 7H9 medium supplemented with 10% OADC (Oleic Acid, Albumin, Dextrose, Catalase). Exponential growth phase culture was harvested at OD_600_ between 0.5 and 0.8 and washed twice in PBS. The small-size RNA (approx. <200 nucleotides) enriched fraction was extracted using the mirVana™ miRNA Isolation Kit (Ambion, Austin, TX, USA) according to manufacturer’s instruction. Both small-size RNA enriched and total RNA (depleted sRNAs) fractions were collected and analysed with Agilent 2100 Bioanalyzer (Agilent Biotechnologies, Santa Clara, CA, USA).

### Candidate sRNAs, Promoter and Terminator Consensus Sequences

A custom analysis pipeline was developed for analyzing Illumina reads (details are reported in Pellin *et al.* [22]): after computational processing of reads to remove the poly-A tail, reads have been mapped to the whole genome using SOAPv1 tool, and those regions coding for proteins or for functional RNA molecules (tRNAs and rRNAs) were excluded from the analysis. To identify putative loci encoding for sRNAs a coverage map and a conservation map were superimposed. Coverage value of a specific genome base derives from RNA-seq data and corresponds to the count of reads overlapping that specific position. The calculation of the conservation value is based on the analysis of a BLASTN 2.0 output file, regarding the pairwise alignment of each MTB intergenic sequences and a database containing all intergenic sequences extracted from all the others genomes in the MTB genus. The conservation value then corresponds to the weighted count of genomes within the MTB genus, with at least one pairwise alignment hit that satisfies high quality criteria.

To identify sRNA candidates the following criteria were considered:

1- region coverage by consecutive nucleotides;

2- region length ≥30 and ≤550 nucleotides.

And at least one of the following conditions must be satisfied:

3.1 RNA-seq coverage value ≥50 or,

3.2 Genomic region conservation value ≥1.77 or,

3.3 RNA-seq coverage value ≥22 and genomic region conservation value ≥0.97.

Coverage and conservation thresholds were established on the empirical distribution functions and determined by means of statistical criteria according to data characteristics.

In order to assess candidates’ reliability, many different characteristics of each putative sRNA were analyzed. In particular, Minimum Folding Energy (MFE) and relative p-value (MFE p-value) were taken into account.

The application of such pipeline (fully described in [22]) to our data resulted in the identification of 1948 sRNA candidates, classified as Type A (based on criteria 1, 2 and 3.1; 1378 candidates), Type B (based on criteria 1,2 and 3.3; 114 candidates) and Type C (based on criteria 1,2 and 3.2; 456 candidates). In this work we focused on Type A candidates only. In fact, we would expect that the most promising candidates are included in this category, whereas Type B and Type C could include higher number of false positives due to the criteria used for their identification.

For the present work, an update on the annotation of the candidates has been performed in January 2012 to include in the genome most recently annotated CDSs [53]. In particular, the analysis published in Pellin *et al.* [22] was performed using as reference the NC_000962.gff database; whereas for the update the NC_000962.2.gff database was used. Candidates obtained by RNA-seq were further analyzed for the presence of promoter and/or terminator consensus sequences. For the analysis of the promoter region we used the consensus sequences described by Sachdeva and coworkers [54]. In particular, we analyzed the region between −50 and +15 nucleotides from the predicted 5′-end of the candidates for the presence of a Sigma A (sigA) −10 consensus motif (mismatch: 0). In particular, we analyzed the region between −50 and +15 nucleotides from the predicted 5′-end of the candidates for the presence of a Sigma A (sigA) −10 consensus motif TANNNT (with allow mismatch equal to 0) by means of a custom script written in *R* and using BioConductor package Biostrings [55].

In order to identify putative terminators, we applied both GeSter and RNIE algorithm.

Gester analysis was performed by means of the web based version called WebGester [56] with parameters set to default. Results reported in this paper regards to putative terminators satisfying ΔG cut-off equal to −16.98.

The RNIE algorithm was run in the “gene mode” that is optimized to individually annotate the downstream regions of genes or, more generally, putative encoding regions. We followed the same pipeline suggested by RNIE authors in [25]. Initially we took all the annotated coding sequences from the MTB complete genome and extracted subsequences from −20 to +80 nt around all annotated gene termini, according to available annotation file. Each of these was folded using the RNAfold routine from the Vienna package [57] and then subjected to a permutation test where native MFEs were compared to the pooled distribution of MFEs for 1000 permuted sequences with the same dinucleotide content in each termini. The regions that had a *P*<0.001 where subsequently fed into the alignment and folding algorithm Cmfinder [58]. The three most significant covariance model obtained from MTB CDS and TRIT model proposed in [25] were deployed to annotate the downstream regions of putative candidates.

### Microarray for sRNA Candidates Expression

CombiMatrix microarray technology (Irvine, CA, USA) has been adopted to validate putative sRNA candidates by expression evidence. Probe sets for candidates have been designed according to the following criteria: the probes were non-overlapping sequences of 30 nt, spanning all the candidate length. Probes were located at least 5 nt from each other and from the candidate boundaries. As result, probe sets were made of one single probe when candidates were shorter than 75 nt of length; two probes when candidates length were between 75 and 110 nt; three probes when candidates length were between 110 and 180 nt, and four probes for longer candidates. To each probe, a control mismatch probe was provided (mismatch: two bases). As positive controls, probes targeting the following genes have been included: *sigA* (*Rv2703*), *16S* (*rrs, Rvnr01*), and 5S (*rrf, Rvnr03*). As negative controls we considered the same number of probes of the positive controls, made of random sequences not mapping in the *M. tuberculosis* H37Rv genome. Each array contained two complete probe sets and relative mismatches, each in duplicate. Small-size RNA fraction and total RNA fraction depleted of small-size RNAs were prepared using mirVana miRNA Isolation kit (Applied Biosystems/Ambion, Austin, TX, USA). The ULS labeling kit for CombiMatrix arrays (Kreatech Biotechnology BV, Amsterdam, The Netherlands) was used to label RNA samples with biotin and for hybridization on a 12K array followed by electrochemical detection on the ElectraSense reader performed according to manufacturer’s instructions. Two independent experiments were carried out starting from two biological replicates each (*M. tuberculosis* H37Rv grown in Middlebrook 7H9 10% OADC, OD_600_ between 0.5 and 0.8). Microarray hybridization was carried out twice (technical replicate) for each biological replicate; every microarray was acquired at least twice. Results were analyzed applying Robust Multichip Analysis (RMA) procedure on the logarithm of the background (mismatches) corrected perfect match electrical current intensities.

We compared the candidates expression with controls by means of a t-type statistic test. P-values were computed by means of permutations, to avoid any distributional assumption, and adjusted for multiplicity by means of the Holm-Bonferroni method [59], [60]. Candidates showing an expression mean significantly higher than the expression of controls (p-value <0.05) were considered as validated. Then, a combined p-value (namely p-adj) for each candidate was calculated with Fisher rule considering both RNA-seq and microarray results.

### Criteria for the Classification of sRNAs

We decided to consider approximately 60 nucleotides at the 5′ end of a gene as putative promoter region. As reported by Gardner and colleagues [25], the majority of termination signals are within 80 nucleotides from the 3′-end of a gene. Thus, we have considered as intergenic those sRNAs mapping more than 80 nt upstream or downstream of a neighboring CDSs. Candidates mapping within ≤80 nucleotides from a CDS on the same strand were considered potentially synthesized as untranslated regions (5′/3′ UTRs). Candidates synthesized by the strand opposite to a CDS or a UTR are antisense (AS) sRNAs, and can be divided in subclasses depending on their relative position.

### Northern Blot Analysis

10 µg of total RNA were separated on a 6% denaturing polyacrilamyde gel, blotted on a positively charged membrane (Hybond N+, GE Life Sciences), and hybridized to specific ^32^P-labeled riboprobes or oligonucleotides, as described previously [61]. Membranes were exposed to a phosphor screen and visualized with a phosphorimager. The oligonucleotides used are reported in Table S4 and Table S5, respectively.

### Primer Extension Analysis

The oligonucleotide probes were 5′ end labeled with T4 polynucleotide kinase (Promega). 5′ ends were identified by primer extension analysis, as described in Boorstein and Craig [62]: an annealing mix (10 µl), containing 10 µg of total RNA, 1 U/µl RNasin (Promega), 0.5 pmole radiolabeled oligo and 1X ss-hybridization buffer (300 mM NaCl, 10 mM Tris HCl pH 7.5, 2 mM EDTA), was denatured at 80°C for 4 minutes and incubated for 2 hours at 50°C for the annealing. Then, to the annealing mix were added 40 µl of 1.25 X RT-buffer (1.25 mM of each dNTP, 12.5 mM DTT, 12.5 mM Tris HCl pH 8, 7.5 mM MgCl_2_), 5 U RNasin (Promega) and 10 U AMV Reverse Transcriptase (Finnzymes) and incubated 30 min at 50°C for the extension. RNA was hydrolyzed with NaOH and all samples were neutralized with HCl and then precipitated and dissolved in 6 µl stop mix (95% formamide, 20 mM EDTA, 0.05% bromophenol blue, 0.05% xylene cyanol FF). Reaction products were separated on a 6% denaturing polyacrylamide gel along with sequencing reactions made with the same labeled oligonucleotides used for the primer extensions.

### Statistical Analysis of the −10 Region

We used a statistical approach to further validate sRNA candidates. The approach described by Dornenburg [23] was adapted for this scope.

In summary, we compared the frequency of specific nucleotides at fixed positions within the −10 consensus region. Due to the lack of consistent published data on −10 consensus sequences in MTB, we considered only the TANNNT hexamer recognized by the sigma factor sigA. Therefore, we analyzed the frequency of T in position −13, A in position −12 and T in position −8 for our sRNA candidates, for annotated CDSs in the genome of MTB and for a set random sequences, all of the same sample size. Random sequences were generated and used as control. A score of 3 was attributed when all the 3 nucleotides TAT were found at the expected position (−13, −12, −8, respectively); a score of 2, 1 or 0 was attributed when 2, 1 or 0 nucleotides, respectively were found at expected positions. An average of the scores was then calculated for each of the three categories (candidates, annotated CDSs and control sequences). A Mann-Whitney test statistic was used to compare scores and p-values computed by permutation methods. For comparison we considered a number of random sequences equal to the sample size considered by the permutation. Sample sizes were as follow: 1948 candidates (all), 1373 type A candidates, 260 validated candidates, 4048 CDSs. All statistical analyses were performed in R and Bioconductor environment [55].

### Functional Enrichment of Antisense Regulated Genes

We obtained functional annotation for MTB genes from three different sources: (*i*) GO terms from the DAVID database [63], (*ii*) metabolic pathways from the KEGG database [64], and (*iii*) functional categories from the TubercuList database (http://genolist.pasteur.fr/TubercuList). We then applied the Fisher’s Exact Test to determine biological terms that are enriched among antisense-regulated genes, based on the contingency table shown in Table S6. The test was slightly modified by removing one gene from the list of interest (i.e. *a-1*) before computing the Fisher exact probability; this modification removes the effect of terms based on single genes only [65].

## Supporting Information

Figure S1 **Schematic representation of sRNA candidates classification according to their mapping position.** Thick black arrows indicate coding sequences (CDS); thin arrows indicate sRNAs; dotted arrows the distance from the 5′/3′ ends of CDS to the putative 5′/3′end of the sRNA; AS: antisense.
(TIF)

Figure S2 **Schematic representation of the distance between the −10 consensus hexamer in candidate sRNAs, annotated coding region and random sequences.** Picture of the relative mean score differences among sequences groups. To preserve as best as possible these dissimilarity, we obtained the plot coordinates from a non-linear multidimensional scaling of the pairwise absolute differences, taken as inter-groups distance matrix.
(TIF)

Figure S3 **Schematic representation of the mapping position of sRNA candidates validated by Northern blot (from 54 to 1029).** Empty arrows represent coding sequences (CDS), whereas sRNA candidates are reported as grey arrows. Dotted arrow (candidate 1359) represents the possibly erroneous annotation of the CDS Rv2165c. Dotted lines represent UTRs.
(TIF)

Figure S4 **Schematic representation of the mapping position of sRNA candidates validated by Northern blot (from 1080 to 1565).**
(TIF)

Figure S5 **Northern blot for candidate 1938 in** ***M. tuberculosis*** **H37Rv and H37Ra (exponential growth phase).**
(TIF)

Figure S6 **Schematic representation for the mapping position of the sRNA 1359 validated by Northern blot.**
(TIF)

Table S1 **Dataset including all the candidates (A, B, C types) representing the starting point of the present work.**
(XLSX)

Table S2 **Functional categories of genes showing RNA species within the 5′ UTR.** Classification was performed according to TubercuList.
(XLSX)

Table S3 **Functional enrichment of** ***cis-*** **regulated genes.**
(XLS)

Table S4 **List of oligonucleotides used in the Northern blot.** * Oligo: ^32^P-labelled oligonucleotides used as probes in the Northern blot assays; FWD or REV: primers used to amplify the genomic regions to be used as templates in the *in vitro* transcription of the riboprobes.
(XLSX)

Table S5 **List of other oligonucleotides used for the primer extension.** DAVID database and TubercuList have been used for functional annotation of MTB genes. GO: gene onthology; mtu: KEGG pathway. TBL: TubercuList.
(XLSX)

Table S6 **Contingency table for evaluating functional enrichment of antisense-regulated genes.** a = number of AS-regulated genes annotated with term j; b = number of AS-regulated genes not annotated with term j. The quantities c, and d are similarly defined for the remaining genes not overlapped by AS sRNAs. N is the total number of *M. tuberculosis* genes for which annotation is available.
(XLSX)
