# Supplementary figures and images for: Genome-Wide Discovery of Small RNAs in Mycobacterium tuberculosis
Source: PLoS One. 2012 Dec 19;7(12):e51950. doi: 10.1371/journal.pone.0051950 (PMC3526491; doi:10.1371/journal.pone.0051950)

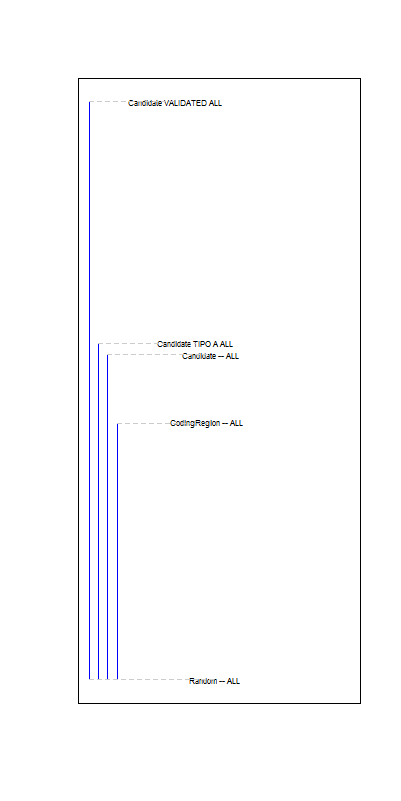

Supplement: Figure S2 — Schematic representation of the distance between the −10 consensus hexamer in candidate sRNAs, annotated coding region and random sequences. Picture of the relative mean score differences among sequences groups. To preserve as best as possible these dissimilarity, we obtained the plot coordinates from a non-linear multidimensional scaling of the pairwise absolute differences, taken as inter-groups distance matrix. (TIF) [file pone.0051950.s002.tif]

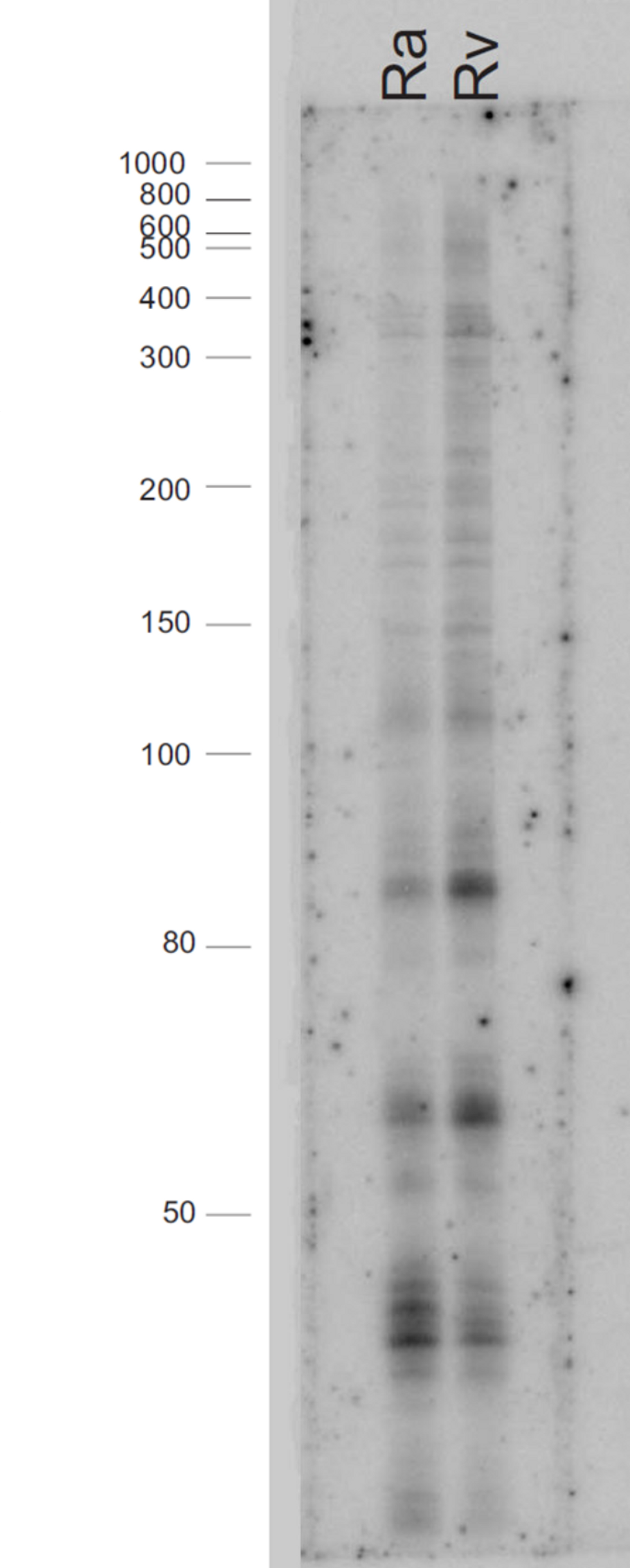

Supplement: Figure S5 — Northern blot for candidate 1938 in M. tuberculosis H37Rv and H37Ra (exponential growth phase). (TIF) [file pone.0051950.s005.tif]
